# Crystal structure and Hirshfeld surface analysis of 3,4-dihydro-2-(2,4-dioxo-6-methylpyran-3-ylidene)-4-(4-pyridin-4-yl)-1,5-benzodiazepine

**DOI:** 10.1107/S2056989018017565

**Published:** 2019-01-01

**Authors:** Lhoussaine El Ghayati, Youssef Ramli, Tuncer Hökelek, Mohamed Labd Taha, Joel T. Mague, El Mokhtar Essassi

**Affiliations:** a Laboratoire de Chimie Organique Hétérocyclique URAC 21, Pôle de Compétence Pharmacochimie, Av. Ibn Battouta, BP 1014, Faculté des Sciences, Université Mohammed V, Rabat, Morocco; b Laboratory of Medicinal Chemistry, Faculty of Medicine and Pharmacy, Mohammed V University, Rabat, Morocco; c Department of Physics, Hacettepe University, 06800 Beytepe, Ankara, Turkey; d Laboratoire de Chimie Bioorganique Appliquée, Faculté des Sciences, Université Ibn Zohr, Agadir, Morocco; e Department of Chemistry, Tulane University, New Orleans, LA 70118, USA

**Keywords:** crystal structure, hydrogen bond, π-stacking, benzodiazepine, Hirshfeld surface

## Abstract

The title compound, C_20_H_17_N_3_O_3_ [systematic name: 2-(6-methyl-2,4-dioxo­pyran-3-yl­idene)-4-(pyridin-4-yl)-2,3,4,5-tetra­hydro-1*H*-1,5-benzodiazepine], is built up from a benzodiazepine ring system linked to pyridyl and pendant di­hydro­pyran rings, where the benzene and pyridyl rings are oriented at a dihedral angle of 43.36 (6)°. The pendant di­hydro­pyran ring is rotationally disordered in a 90.899 (3):0.101 (3) ratio with the orientation of each component largely determined by intra­molecular N—H_Diazp_⋯O_Dhydp_ (Diazp = diazepine and Dhydp = di­hydro­pyran) hydrogen bonds. In the crystal, mol­ecules are linked via pairs of weak inter­molecular N—H_Diazp_⋯O_Dhydp_ hydrogen bonds, forming inversion-related dimers with *R*_2_^2^(26) ring motifs. The dimers are further connected along the *b*-axis direction by π–π stacking inter­actions between the pendant di­hydro­pyran and pyridyl rings with centroid–centroid distances of 3.833 (3) Å and a dihedral angle of 14.51 (2)°. Hirshfeld surface analysis of the crystal structure indicates that the most important contributions for the crystal packing are from H⋯H (50.1%), H⋯C/C⋯H (17.7%), H⋯O/O⋯H (16.8%), C⋯C (7.7%) and H⋯N/N⋯H (5.3%) inter­actions. Hydrogen-bonding and van der Waals inter­actions are the dominant inter­actions in the crystal packing.

## Abstract 2

In the title compound, the pendant di­hydro­pyran ring is rotationally disordered in a 90.899 (3):0.101 (3) ratio with the orientation of each component largely determined by intra­molecular N—H⋯O hydrogen bonds. In the crystal, inversion-related mol­ecules form dimers through inter­molecular N—H⋯O hydrogen bonds with the dimers associated along the *b*-axis direction by slipped π-stacking inter­actions between the pyridyl and di­hydro­pyran rings.

## Chemical context   

Diversely substituted 1,5-benzodiazepines and their derivatives embedded with a variety of functional groups are important biological agents and a significant amount of research activity has been directed towards this class of compounds. In fact, many 1,5-benzodiazepines are best known to possess biologically diverse activities such as anti-inflammatory, hypnotic, anti-HIV-1, anti­convulsant and anti­microbial (Roma *et al.*, 1991 ▸; Kalkhambkar *et al.*, 2008 ▸; Kudo, 1982 ▸; De Sarro *et al.*, 1996 ▸; Kumar & Joshi, 2007 ▸). Various methods have been worked out for their synthesis (Dardouri *et al.*, 2011 ▸; Chkirate *et al.*, 2018 ▸; Sebhaoui *et al.*, 2017 ▸). Benzodiazepine derivatives also find commercial use as dyes for acrylic fibers. The search for new heterocyclic systems including the 1,5-benzodiazepine moiety for their biological activities is therefore of much current importance (Tjiou *et al.*, 2005 ▸; Keita *et al.*, 2003 ▸; Jabli *et al.*, 2009 ▸). In this context, we report herein the synthesis, the mol­ecular and crystal structures along with the Hirshfeld surface analysis of the title compound.
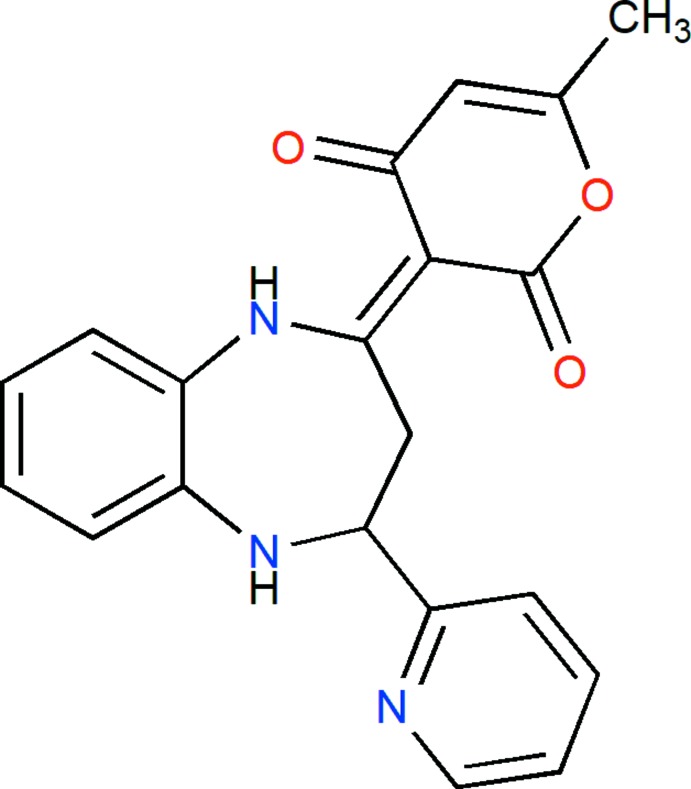


## Structural commentary   

**Figure 1:**
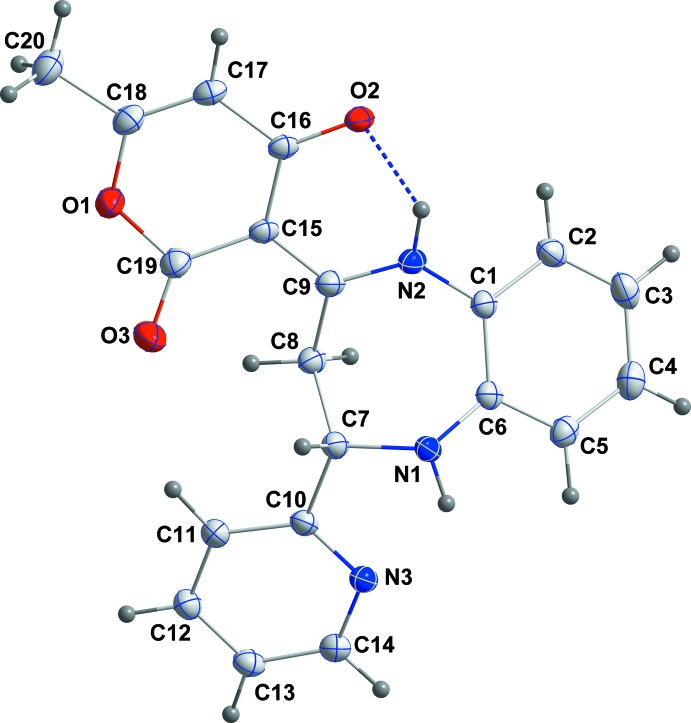
The title mol­ecule with the labelling scheme and 50% probability ellipsoids. Only the major orientation of the disordered di­hydro­pyran ring is shown.

The title compound, (I), is built up from a benzodiazepine ring system linked to pyridyl and pendant di­hydro­pyran rings (Fig. 1 ▸). The benzene ring *A* (C1–C6) is oriented at a dihedral angle of 43.36 (6)° with respect to the pyridyl ring *C* (N3/C10–C14). The pendant di­hydro­pyran ring *D* (O1/C15–C19) shows a 90.899 (3):0.101 (3) disorder with the minor component rotated by 174.6 (4)° from the orientation of the major component. The orientation of both components is largely determined by intra­molecular N2—H2*A*⋯O2 or N2—H2*A*⋯O3*A* hydrogen bonds (Table 1 ▸ and Fig. 1 ▸). A puckering analysis of the major orientation of the pendant di­hydro­pyran ring *D* gave the parameters *Q* = 0.127 (2) Å, θ = 108.0 (8)° and φ = 79.6 (8)° while for the seven-membered diazepine ring *B* (N1/N2/C1/C6–C9), the parameters are *Q*(2) = 0.8888 (13) Å, *Q*(3) = 0.2070 (13) Å, φ(2) = 201.03 (8)° and φ(3) = 293.9 (4)°.

## Supra­molecular features   

**Figure 2:**
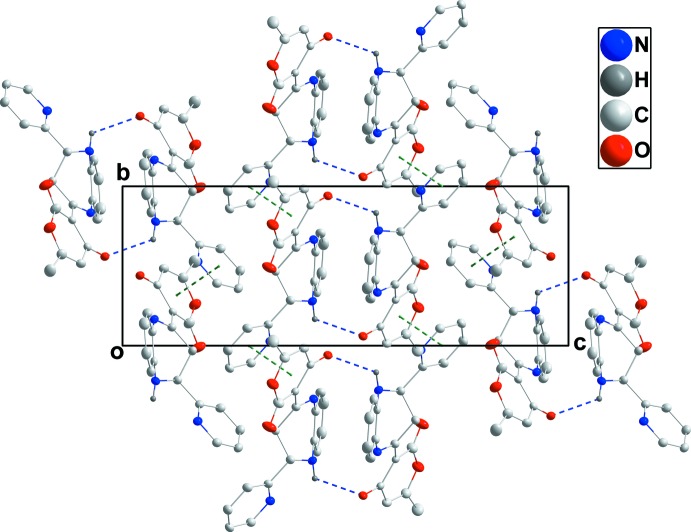
Packing viewed along the *a*-axis direction. The inter­molecular N—H_Diazp_⋯O_Dhydp_ (Diazp = diazepine and Dhydp = di­hydro­pyran) hydrogen bonds and slipped π–π stacking inter­actions are shown, respectively, by blue and green dashed lines.

In the crystal, the mol­ecules are linked via pairs of weak inter­molecular N—H_Diazp_⋯O_Dhydp_ (Diazp = diazepine and Dhydp = di­hydro­pyran) hydrogen bonds (Table 1 ▸), forming inversion-related dimers with 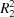
(26) ring motifs. The dimers are further connected along the *b*-axis direction (Fig. 2 ▸) by π–π-stacking inter­actions between the pendant di­hydro­pyran and pyridyl rings [*Cg*1⋯*Cg*2 (*x*, 1 + *y*, *z*) = 3.833 (3) Å with a dihedral angle of 14.51 (2)°; *Cg*1 and *Cg*2 are the centroids of rings *D* (O1/C15–C19) and *C* (N3/C10–C14), respectively].

## Hirshfeld surface analysis   

**Figure 3:**
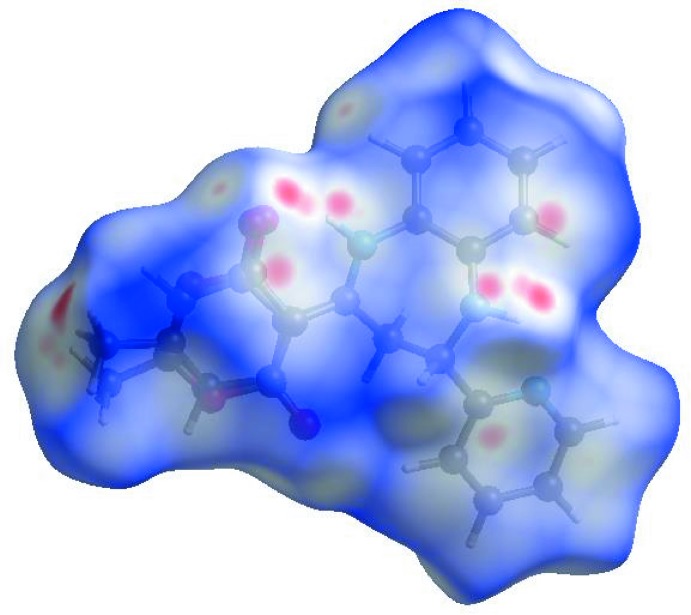
View of the three-dimensional Hirshfeld surface of the title compound plotted over *d*_norm_ in the range −0.2111 to 1.1395 a.u.

**Figure 4:**
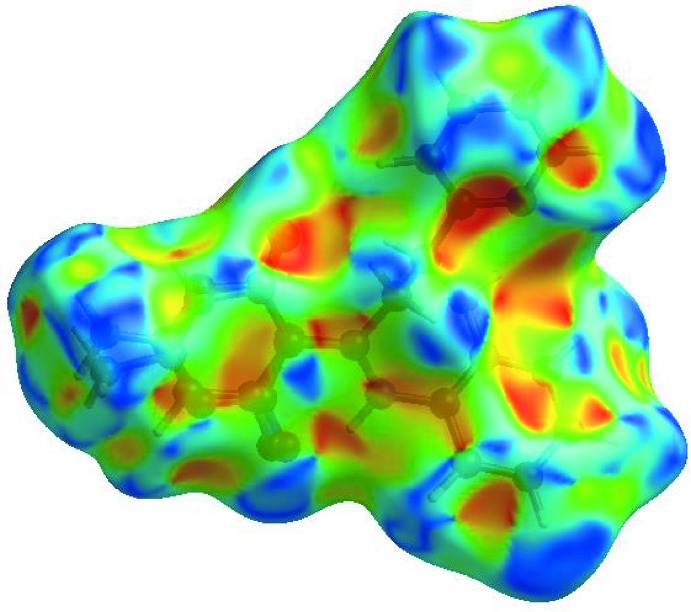
Hirshfeld surface of the title compound plotted over shape-index.

**Figure 5:**
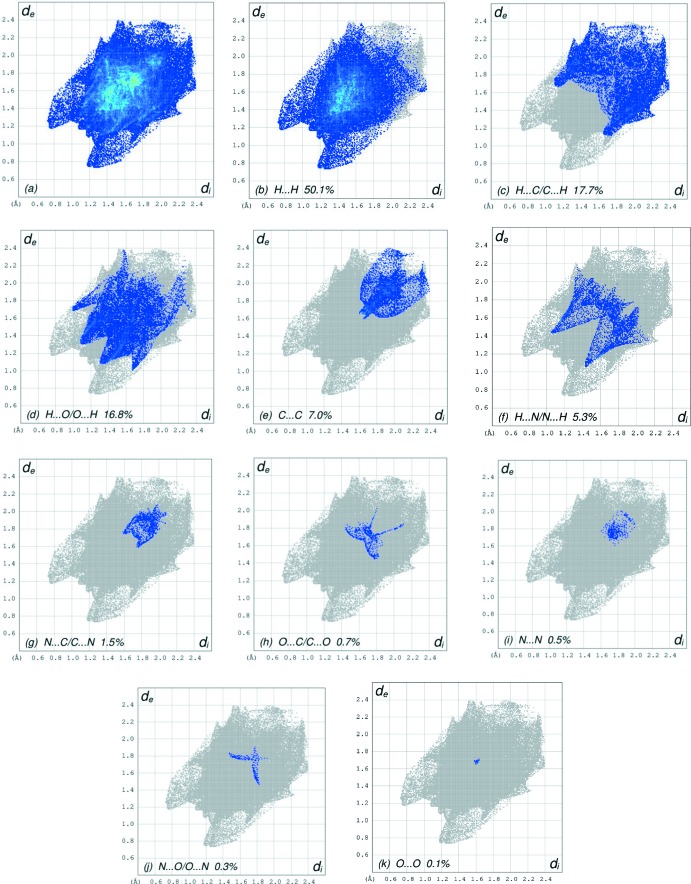
The full two-dimensional fingerprint plots for the title compound, showing (*a*) all inter­actions, and delineated into (*b*) H⋯H, (*c*) H⋯C/C⋯H, (*d*) H⋯O/O⋯H, (*e*) C⋯C, (*f*) H⋯N/N⋯H, (*g*) N⋯C/C⋯N, (*h*) O⋯C/C ⋯ O, (i) N⋯N, (*j*) N⋯O/O⋯N and (*k*) O⋯O inter­actions. The *d*_i_ and *d*_e_ values are the closest inter­nal and external distances (in Å) from given points on the Hirshfeld surface contacts.

In order to visualize the inter­molecular inter­actions in the crystal of the title compound, a Hirshfeld surface (HS) analysis (Hirshfeld, 1977 ▸; Spackman & Jayatilaka, 2009 ▸) was carried out by using *CrystalExplorer17.5* (Turner *et al.*, 2017 ▸). In the HS plotted over *d*_norm_ (Fig. 3 ▸), the white area indicates contacts with distances equal to the sum of van der Waals radii, and the red and blue areas indicate distances shorter (in close contact) or longer (distinct contact), respectively, than the van der Waals radii (Venkatesan *et al.*, 2016 ▸). The bright-red spots appearing near O2 and hydrogen atoms H1 and H2*A* indicate their roles as the respective donors and/or acceptors in the dominant N—H⋯O hydrogen bonds. The shape-index of the HS is a tool for visualizing the π–π stacking by the presence of adjacent red and blue triangles; if there are no adjacent red and/or blue triangles, then there are no π–π inter­actions. Fig. 4 ▸ clearly suggest that there are π–π inter­actions in (I). The overall two-dimensional fingerprint plot, Fig. 5 ▸(*a*), and those delineated into H⋯H, H⋯C/C⋯H, H⋯O/O⋯H, C⋯C, H⋯N/N⋯H, N⋯C/C⋯N, O⋯C/C⋯O, N⋯N, N⋯O/O⋯N and O⋯O contacts (McKinnon *et al.*, 2007 ▸) are illustrated in Fig. 5 ▸(*b*)–(*k*), respectively, together with their relative contributions to the Hirshfeld surface. H⋯H inter­actions are the most important, contributing 50.1% to the overall crystal packing, and are shown in Fig. 5 ▸(*b*) as widely scattered points of high density because of the large hydrogen content of the mol­ecule. The two pairs of thin and thick spikes with the tips at *d*_e_ + *d*_i_ ∼2.27 and 1.95 Å, respectively, in Fig. 5 ▸(*b*) are due to the short inter­atomic H⋯H contacts (Table 2 ▸). In the absence of C—H⋯π inter­actions in the crystal, the pair of characteristic wings in the fingerprint plot delineated into H⋯C/C⋯H contacts (17.7% contribution to the HS) have a symmetrical distribution of points, Fig. 5 ▸(*c*), with the tips at *d*_e_ + *d*_i_ ∼2.82 Å. The two pairs of thin and thick spikes with the tips at *d*_e_ + *d*_i_ = 2.67 and 2.40 Å, respectively, in Fig. 5 ▸(*d*) are due to the N—H⋯O hydrogen bonds (Table 1 ▸), as well as the short inter­atomic H⋯O/O⋯H contacts (Table 2 ▸). The C⋯C [Fig. 5 ▸(*e*)] contacts contribute 7.0% to the HS and have symmetrical distribution of points, with the tips at *d*_e_ + *d*_i_ = 3.24 Å. The pair of characteristic wings in the fingerprint plot delineated into H⋯N/N⋯H contacts [5.3% contribution; Fig. 5 ▸(*f*)] has a pair of spikes with the tips at *d*_e_ + *d*_i_ = 1.49 Å. Finally, the N⋯C/C⋯N contacts [Fig. 5 ▸(*g*)] contribute 1.5% to the HS and are viewed as a symmetrical distribution of points with pairs of thin edges at *d*_e_ + *d*_i_ = 3.36 Å.

**Figure 6:**
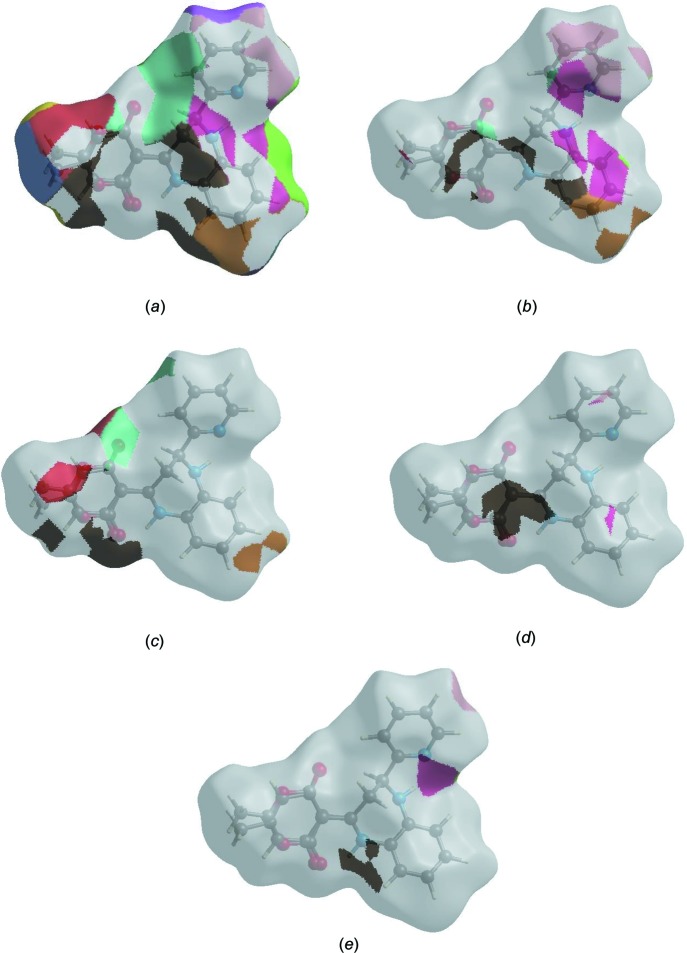
The Hirshfeld surface representations with the function *d*_norm_ plotted onto the surface for (*a*) H⋯H, (*b*) H⋯C/C⋯H, (*c*) H ⋯ O/O⋯H, (*d*) C⋯C and (*e*) H⋯N/N⋯H inter­actions.

The Hirshfeld surface representations with the function *d*_norm_ plotted onto the surface are shown for the H⋯H, H⋯C/C⋯H, H⋯O/O⋯H, C⋯C and H⋯N/N⋯H inter­actions in Fig. 6 ▸(*a*)–(*e*), respectively.

The Hirshfeld surface analysis confirms the importance of H-atom contacts in establishing the packing. The large number of H⋯H, H⋯C/C⋯H, H⋯O/O⋯H and H⋯N/N⋯H inter­actions suggest that van der Waals inter­actions and hydrogen bonding play the major roles in the crystal packing (Hathwar *et al.*, 2015 ▸).

## Synthesis and crystallization   

To a suspension of 3-[1-(2-amino­phenyl­imino)­eth­yl]-4-hy­droxy-6-methyl­pyran-2-one (4 mmol) in ethanol (40 ml) were added 1.5 equivalents of 2-pyridine­carboxaldehyde and three drops of tri­fluoro­acetic acid (TFA). The mixture was refluxed for 4 h. Cooling to room temperature induced the precipitation of a yellow solid, which was filtered off and washed with 20 ml of cold ethanol. Cooling to room temperature induced the precipitation of a yellow solid, which was filtered and washed with 20 ml of cold ethanol. Crystals suitable for X-ray analysis were obrained by recrystallization of the product from ethanol solution.

## Refinement   

Crystal data, data collection and structure refinement details are summarized in Table 3 ▸. The pendant di­hydro­pyran ring is rotationally disordered in a 90.899 (3):0.101 (3) ratio. As a result of this disorder, the hydrogen atoms on C17 and C20 and their disordered counterparts were placed in calculated positions and included as riding contributions. The alternate orientation of this ring was treated as a rigid group having the same geometry as the major component. The remaining H atoms were located in a difference-Fourier map and were freely refined.

## Supplementary Material

Crystal structure: contains datablock(s) I, global. DOI: 10.1107/S2056989018017565/lh5888sup1.cif

Structure factors: contains datablock(s) I. DOI: 10.1107/S2056989018017565/lh5888Isup2.hkl

Supporting information file. DOI: 10.1107/S2056989018017565/lh5888Isup3.cdx

Supporting information file. DOI: 10.1107/S2056989018017565/lh5888Isup4.cml

CCDC reference: 1884597

Additional supporting information:  crystallographic information; 3D view; checkCIF report

## Figures and Tables

**Table 1:** Hydrogen-bond geometry (Å, °)

*D*—H⋯*A*	*D*—H	H⋯*A*	*D*⋯*A*	*D*—H⋯*A*
N1—H1⋯O2^iii^	0.889 (18)	2.536 (18)	3.089 (2)	121.0 (14)
N2—H2*A*⋯O2	0.928 (18)	1.836 (18)	2.616 (2)	140.1 (15)
N2—H2*A*⋯O3*A*	0.928 (18)	1.58 (2)	2.382 (15)	142.0 (17)

Symmetry code: (iii) 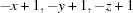.

**Table 2:** Selected interatomic distances (Å)

O2⋯C7^i^	3.22	N3⋯H1	2.330 (16)
O2⋯N1^i^	3.25	N3⋯H13^vii^	2.755 (17)
O2⋯N2	2.62	C5⋯C16^iii^	3.40
O2⋯C2^ii^	3.40	C7⋯C16^vi^	3.60
O2⋯C10^i^	3.29	C9⋯C14^i^	3.38
O2⋯N1^iii^	3.09	C10⋯C16^vi^	3.31
O2⋯C5^iii^	3.23	C11⋯C16^vi^	3.52
O3⋯C8	2.85	C11⋯C17^vi^	3.49
O3⋯C7	3.37	C1⋯H8*B*	2.547 (17)
O1⋯H11^iv^	2.84	C4⋯H20*A* ^viii^	3.09
O2⋯H1^i^	2.85	C5⋯H20*A* ^viii^	2.98
O2⋯H1^iii^	2.54	C6⋯H8*B*	2.548 (17)
O2⋯H2*A*	1.84	C8⋯H11	2.930 (16)
O2⋯H5^iii^	2.86	C11⋯H8*A*	2.688 (15)
O2⋯H7^i^	2.75	C13⋯H8*B* ^ix^	2.895 (18)
O2⋯H2^ii^	2.62	C14⋯H8*B* ^ix^	2.855 (17)
O3⋯H8*A*	2.25	C16⋯H2*A*	2.40
O3⋯H11	2.73	C17⋯H7^i^	2.92
O3⋯H12^iv^	2.69	C19⋯H8*A*	2.60
O3⋯H20*C* ^v^	2.71	H1⋯H5	2.29 (2)
N1⋯O2^vi^	3.25	H1⋯H2*A* ^iii^	2.50 (2)
N1⋯N2	2.909 (3)	H2⋯H2*A*	2.41 (3)
N1⋯N3	2.727 (3)	H3⋯H4^x^	2.57 (3)
N1⋯O2^iii^	3.09	H5⋯H20*A* ^viii^	2.4596
N1⋯N2^iii^	3.078 (3)	H7⋯H17^vi^	2.58
N2⋯O2	2.62	H8*A*⋯H11	2.31 (2)
N2⋯C6^iii^	3.319 (3)	H8*A*⋯H20*C* ^v^	2.50
N1⋯H2*A* ^iii^	2.547 (17)	H17⋯H20*A*	2.47

Symmetry codes: (i) 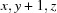; (ii) 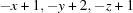; (iii) 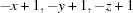; (iv) 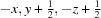; (v) 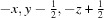; (vi) 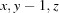; (vii) 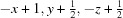; (viii) 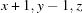; (ix) 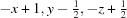; (x) 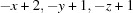.

**Table 3:** Experimental details

Crystal data	
Chemical formula	C_20_H_17_N_3_O_3_
*M*_r_	347.36
Crystal system, space group	Monoclinic, *P*2_1_/*c*
Temperature (K)	100
*a*, *b*, *c* (Å)	10.509 (9), 7.435 (6), 21.367 (16)
β (°)	103.041 (15)
*V* (Å^3^)	1626 (2)
*Z*	4
Radiation type	Mo *K*α
μ (mm^−1^)	0.10
Crystal size (mm)	0.31 × 0.23 × 0.21

Data collection	
Diffractometer	Bruker SMART APEX CCD
Absorption correction	Multi-scan (*SADABS*; Krause *et al.*, 2015 ▸)
*T*_min_, *T*_max_	0.84, 0.98
No. of measured, independent and observed [*I* > 2σ(*I*)] reflections	30100, 4368, 3541
*R*_int_	0.038
(sin θ/λ)_max_ (Å^−1^)	0.685

Refinement	
*R*[*F* ^2^ > 2σ(*F* ^2^)], *wR*(*F* ^2^), *S*	0.048, 0.135, 1.07
No. of reflections	4368
No. of parameters	294
No. of restraints	1
H-atom treatment	H atoms treated by a mixture of independent and constrained refinement
Δρ_max_, Δρ_min_ (e Å^−3^)	0.45, −0.20

Computer programs: *APEX3* and *SAINT* (Bruker, 2016 ▸), *SHELXT* (Sheldrick, 2015*a* ▸), *SHELXL2018* (Sheldrick, 2015*b* ▸), *Mercury* (Macrae *et al.*, 2008 ▸) and *SHELXTL* (Sheldrick, 2008 ▸).

## supplementary crystallographic information

### Crystal data

**Table:**

C_20_H_17_N_3_O_3_	*F*(000) = 728
*M**_r_* = 347.36	*D*_x_ = 1.419 Mg m^−^^3^
Monoclinic, *P*2_1_/*c*	Mo *K*α radiation, λ = 0.71073 Å
*a* = 10.509 (9) Å	Cell parameters from 9961 reflections
*b* = 7.435 (6) Å	θ = 2.5–29.1°
*c* = 21.367 (16) Å	µ = 0.10 mm^−^^1^
β = 103.041 (15)°	*T* = 100 K
*V* = 1626 (2) Å^3^	Block, orange
*Z* = 4	0.31 × 0.23 × 0.21 mm

### Data collection

**Table:**

Bruker SMART APEX CCD diffractometer	4368 independent reflections
Radiation source: fine-focus sealed tube	3541 reflections with *I* > 2σ(*I*)
Graphite monochromator	*R*_int_ = 0.038
Detector resolution: 8.3333 pixels mm^-1^	θ_max_ = 29.1°, θ_min_ = 2.0°
φ and ω scans	*h* = −14→14
Absorption correction: multi-scan (SADABS; Krause *et al.*, 2015)	*k* = −10→10
*T*_min_ = 0.84, *T*_max_ = 0.98	*l* = −29→29
30100 measured reflections	

### Refinement

**Table:**

Refinement on *F*^2^	Primary atom site location: structure-invariant direct methods
Least-squares matrix: full	Secondary atom site location: difference Fourier map
*R*[*F*^2^ > 2σ(*F*^2^)] = 0.048	Hydrogen site location: mixed
*wR*(*F*^2^) = 0.135	H atoms treated by a mixture of independent and constrained refinement
*S* = 1.07	*w* = 1/[σ^2^(*F*_o_^2^) + (0.0814*P*)^2^ + 0.2932*P*] where *P* = (*F*_o_^2^ + 2*F*_c_^2^)/3
4368 reflections	(Δ/σ)_max_ < 0.001
294 parameters	Δρ_max_ = 0.45 e Å^−^^3^
1 restraint	Δρ_min_ = −0.20 e Å^−^^3^

### Special details

**Table:**

Experimental. The diffraction data were obtained from 3 sets of 400 frames, each of width 0.5 deg. in omega, colllected at phi = 0.00, 90.00 and 180.00 deg. and 2 sets of 800 frames, each of width 0.45 deg in phi, collected at omega = -30.00 and 210.00 deg. The scan time was 15 sec/frame.
Geometry. All esds (except the esd in the dihedral angle between two l.s. planes) are estimated using the full covariance matrix. The cell esds are taken into account individually in the estimation of esds in distances, angles and torsion angles; correlations between esds in cell parameters are only used when they are defined by crystal symmetry. An approximate (isotropic) treatment of cell esds is used for estimating esds involving l.s. planes.
Refinement. Refinement of F^2^ against ALL reflections. The weighted R-factor wR and goodness of fit S are based on F^2^, conventional R-factors R are based on F, with F set to zero for negative F^2^. The threshold expression of F^2^ > 2sigma(F^2^) is used only for calculating R-factors(gt) etc. and is not relevant to the choice of reflections for refinement. R-factors based on F^2^ are statistically about twice as large as those based on F, and R- factors based on ALL data will be even larger. Because of the slight disorder of the dihydropyranone ring, the hydrogen atoms on C17 and C20 and their disordered counterparts were placed in calculated positions and included as riding contributions. The alternate orientation of this ring was treated as a rigid group having the same geometry as the major component.

### Fractional atomic coordinates and isotropic or equivalent isotropic displacement parameters (Å^2^)

**Table:**

	*x*	*y*	*z*	*U*_iso_*/*U*_eq_	Occ. (<1)
N1	0.50328 (10)	0.27877 (14)	0.42584 (5)	0.0216 (2)	
H1	0.5339 (17)	0.167 (2)	0.4311 (8)	0.035 (4)*	
N2	0.46375 (10)	0.66567 (14)	0.42864 (5)	0.0202 (2)	
H2A	0.4561 (17)	0.766 (2)	0.4531 (8)	0.035 (4)*	
N3	0.47709 (10)	0.02629 (13)	0.33122 (5)	0.0210 (2)	
C1	0.58855 (12)	0.58808 (16)	0.43268 (6)	0.0195 (2)	
C2	0.69547 (14)	0.70495 (18)	0.44401 (6)	0.0259 (3)	
H2	0.6776 (16)	0.831 (2)	0.4477 (8)	0.030 (4)*	
C3	0.82095 (14)	0.6385 (2)	0.45086 (7)	0.0303 (3)	
H3	0.8932 (19)	0.721 (3)	0.4594 (9)	0.046 (5)*	
C4	0.83965 (13)	0.4535 (2)	0.44650 (7)	0.0283 (3)	
H4	0.9285 (17)	0.409 (2)	0.4496 (8)	0.034 (4)*	
C5	0.73377 (13)	0.33768 (18)	0.43610 (6)	0.0231 (3)	
H5	0.7506 (15)	0.206 (2)	0.4327 (7)	0.026 (4)*	
C6	0.60577 (12)	0.40117 (16)	0.42916 (5)	0.0189 (2)	
C7	0.38159 (12)	0.28441 (16)	0.37634 (6)	0.0210 (3)	
H7	0.3049 (15)	0.268 (2)	0.3989 (7)	0.023 (4)*	
C8	0.36516 (13)	0.46755 (16)	0.34292 (6)	0.0211 (3)	
H8A	0.2883 (14)	0.4618 (19)	0.3074 (8)	0.018 (3)*	
H8B	0.4473 (17)	0.490 (2)	0.3250 (8)	0.029 (4)*	
C9	0.35388 (12)	0.61587 (16)	0.38909 (6)	0.0208 (3)	
C10	0.37298 (12)	0.13296 (15)	0.32695 (6)	0.0195 (2)	
C11	0.25688 (12)	0.10935 (17)	0.28020 (6)	0.0220 (3)	
H11	0.1812 (15)	0.193 (2)	0.2789 (8)	0.028 (4)*	
C12	0.24929 (13)	−0.02918 (17)	0.23603 (6)	0.0239 (3)	
H12	0.1706 (16)	−0.050 (2)	0.2033 (8)	0.024 (4)*	
C13	0.35724 (13)	−0.14053 (17)	0.24005 (6)	0.0228 (3)	
H13	0.3539 (16)	−0.241 (2)	0.2093 (8)	0.033 (4)*	
C14	0.46719 (13)	−0.10813 (16)	0.28802 (6)	0.0214 (3)	
H14	0.5450 (16)	−0.187 (2)	0.2937 (8)	0.029 (4)*	
O1	0.00669 (11)	0.75916 (18)	0.33696 (7)	0.0284 (3)	0.899 (3)
O2	0.33642 (11)	0.94052 (19)	0.46059 (7)	0.0210 (3)	0.899 (3)
O3	0.0900 (2)	0.4903 (3)	0.32931 (15)	0.0353 (6)	0.899 (3)
C15	0.23602 (14)	0.7124 (2)	0.38727 (7)	0.0179 (3)	0.899 (3)
C16	0.23681 (15)	0.8804 (2)	0.42163 (7)	0.0184 (3)	0.899 (3)
C17	0.11731 (18)	0.9845 (2)	0.40703 (8)	0.0223 (3)	0.899 (3)
H17	0.113329	1.095824	0.428316	0.027*	0.899 (3)
C18	0.01213 (16)	0.9261 (2)	0.36392 (8)	0.0243 (3)	0.899 (3)
C19	0.11368 (19)	0.6417 (3)	0.35030 (13)	0.0260 (3)	0.899 (3)
C20	−0.11056 (16)	1.0286 (2)	0.33797 (8)	0.0328 (4)	0.899 (3)
H20A	−0.105441	1.146502	0.358885	0.049*	0.899 (3)
H20B	−0.185256	0.961717	0.346453	0.049*	0.899 (3)
H20C	−0.121537	1.044791	0.291544	0.049*	0.899 (3)
C15A	0.2351 (8)	0.6865 (16)	0.4003 (7)	0.0179 (3)	0.101 (3)
C16A	0.1172 (11)	0.6489 (15)	0.3522 (8)	0.0184 (3)	0.101 (3)
C17A	0.0191 (9)	0.7874 (16)	0.3410 (7)	0.0223 (3)	0.101 (3)
H17A	−0.059812	0.768813	0.309749	0.027*	0.101 (3)
C18A	0.0373 (7)	0.9429 (13)	0.3741 (5)	0.0243 (3)	0.101 (3)
O1A	0.1528 (8)	0.9856 (13)	0.4151 (5)	0.0284 (3)	0.101 (3)
C19A	0.2584 (7)	0.8647 (17)	0.4267 (6)	0.0260 (3)	0.101 (3)
C20A	−0.0603 (10)	1.0911 (14)	0.3745 (7)	0.0328 (4)	0.101 (3)
H20D	−0.148892	1.041236	0.363612	0.049*	0.101 (3)
H20E	−0.051332	1.182826	0.342868	0.049*	0.101 (3)
H20F	−0.044162	1.145564	0.417348	0.049*	0.101 (3)
O2A	0.0956 (15)	0.5021 (19)	0.3224 (11)	0.0210 (3)	0.101 (3)
O3A	0.3599 (8)	0.926 (2)	0.4586 (9)	0.0353 (6)	0.101 (3)

### Atomic displacement parameters (Å^2^)

**Table:**

	*U*^11^	*U*^22^	*U*^33^	*U*^12^	*U*^13^	*U*^23^
N1	0.0252 (5)	0.0167 (5)	0.0189 (5)	−0.0021 (4)	−0.0032 (4)	0.0025 (4)
N2	0.0239 (5)	0.0173 (5)	0.0180 (5)	0.0024 (4)	0.0017 (4)	−0.0015 (4)
N3	0.0227 (5)	0.0182 (5)	0.0212 (5)	−0.0007 (4)	0.0032 (4)	−0.0006 (4)
C1	0.0216 (6)	0.0193 (5)	0.0159 (5)	0.0002 (4)	0.0006 (4)	−0.0011 (4)
C2	0.0303 (7)	0.0206 (6)	0.0238 (6)	−0.0047 (5)	−0.0004 (5)	−0.0012 (5)
C3	0.0253 (7)	0.0344 (7)	0.0280 (7)	−0.0087 (6)	−0.0007 (5)	0.0005 (6)
C4	0.0222 (7)	0.0373 (7)	0.0235 (6)	0.0011 (5)	0.0013 (5)	−0.0001 (5)
C5	0.0252 (6)	0.0251 (6)	0.0175 (5)	0.0040 (5)	0.0017 (5)	−0.0002 (5)
C6	0.0229 (6)	0.0198 (5)	0.0124 (5)	0.0000 (4)	0.0007 (4)	−0.0004 (4)
C7	0.0234 (6)	0.0179 (5)	0.0190 (5)	0.0003 (4)	−0.0004 (5)	−0.0007 (4)
C8	0.0253 (6)	0.0187 (5)	0.0175 (5)	0.0025 (4)	0.0010 (5)	−0.0015 (4)
C9	0.0258 (6)	0.0168 (5)	0.0188 (5)	0.0009 (4)	0.0027 (5)	−0.0003 (4)
C10	0.0225 (6)	0.0159 (5)	0.0194 (5)	−0.0023 (4)	0.0035 (4)	−0.0006 (4)
C11	0.0212 (6)	0.0194 (6)	0.0239 (6)	−0.0009 (4)	0.0019 (5)	−0.0014 (5)
C12	0.0251 (6)	0.0229 (6)	0.0211 (6)	−0.0012 (5)	0.0001 (5)	−0.0026 (5)
C13	0.0282 (6)	0.0199 (6)	0.0200 (6)	−0.0016 (5)	0.0050 (5)	−0.0032 (4)
C14	0.0236 (6)	0.0190 (5)	0.0219 (6)	0.0001 (5)	0.0058 (5)	−0.0003 (5)
O1	0.0218 (5)	0.0272 (7)	0.0338 (6)	0.0046 (4)	0.0013 (4)	−0.0068 (5)
O2	0.0261 (6)	0.0166 (5)	0.0190 (5)	0.0032 (4)	0.0021 (5)	−0.0011 (4)
O3	0.0278 (6)	0.0271 (7)	0.0482 (13)	−0.0006 (5)	0.0028 (6)	−0.0138 (8)
C15	0.0243 (6)	0.0143 (6)	0.0149 (8)	0.0022 (5)	0.0038 (5)	0.0020 (5)
C16	0.0250 (7)	0.0151 (6)	0.0150 (6)	0.0020 (5)	0.0042 (5)	0.0020 (5)
C17	0.0266 (8)	0.0180 (6)	0.0235 (7)	0.0045 (6)	0.0085 (7)	−0.0001 (5)
C18	0.0242 (7)	0.0244 (7)	0.0253 (7)	0.0058 (6)	0.0078 (6)	−0.0005 (5)
C19	0.0234 (7)	0.0254 (7)	0.0284 (7)	0.0046 (6)	0.0044 (6)	−0.0043 (6)
C20	0.0268 (8)	0.0368 (9)	0.0333 (8)	0.0117 (6)	0.0033 (6)	−0.0006 (7)
C15A	0.0243 (6)	0.0143 (6)	0.0149 (8)	0.0022 (5)	0.0038 (5)	0.0020 (5)
C16A	0.0250 (7)	0.0151 (6)	0.0150 (6)	0.0020 (5)	0.0042 (5)	0.0020 (5)
C17A	0.0266 (8)	0.0180 (6)	0.0235 (7)	0.0045 (6)	0.0085 (7)	−0.0001 (5)
C18A	0.0242 (7)	0.0244 (7)	0.0253 (7)	0.0058 (6)	0.0078 (6)	−0.0005 (5)
O1A	0.0218 (5)	0.0272 (7)	0.0338 (6)	0.0046 (4)	0.0013 (4)	−0.0068 (5)
C19A	0.0234 (7)	0.0254 (7)	0.0284 (7)	0.0046 (6)	0.0044 (6)	−0.0043 (6)
C20A	0.0268 (8)	0.0368 (9)	0.0333 (8)	0.0117 (6)	0.0033 (6)	−0.0006 (7)
O2A	0.0261 (6)	0.0166 (5)	0.0190 (5)	0.0032 (4)	0.0021 (5)	−0.0011 (4)
O3A	0.0278 (6)	0.0271 (7)	0.0482 (13)	−0.0006 (5)	0.0028 (6)	−0.0138 (8)

### Geometric parameters (Å, º)

**Table:**

N1—C6	1.3996 (18)	C12—H12	0.967 (16)
N1—C7	1.4648 (18)	C13—C14	1.3818 (19)
N1—H1	0.889 (18)	C13—H13	0.987 (17)
N2—C9	1.3206 (18)	C14—H14	0.992 (16)
N2—C1	1.4175 (19)	O1—C18	1.364 (2)
N2—H2A	0.928 (18)	O1—C19	1.401 (2)
N3—C10	1.3377 (18)	O2—C16	1.2630 (18)
N3—C14	1.3485 (17)	O3—C19	1.217 (2)
C1—C2	1.3976 (19)	C15—C19	1.447 (2)
C1—C6	1.406 (2)	C15—C16	1.448 (2)
C2—C3	1.385 (2)	C16—C17	1.448 (2)
C2—H2	0.959 (17)	C17—C18	1.341 (2)
C3—C4	1.395 (2)	C17—H17	0.9500
C3—H3	0.96 (2)	C18—C20	1.493 (2)
C4—C5	1.385 (2)	C20—H20A	0.9800
C4—H4	0.980 (17)	C20—H20B	0.9800
C5—C6	1.402 (2)	C20—H20C	0.9800
C5—H5	0.997 (16)	C15A—C19A	1.4397
C7—C8	1.5291 (19)	C15A—C16A	1.4466
C7—C10	1.5319 (18)	C16A—O2A	1.2580
C7—H7	1.036 (16)	C16A—C17A	1.4385
C8—C9	1.5020 (18)	C17A—C18A	1.3459
C8—H8A	0.976 (15)	C17A—H17A	0.9500
C8—H8B	1.033 (17)	C18A—O1A	1.3649
C9—C15A	1.423 (3)	C18A—C20A	1.5066
C9—C15	1.4246 (19)	O1A—C19A	1.4057
C10—C11	1.4026 (19)	C19A—O3A	1.2185
C11—C12	1.3870 (19)	C20A—H20D	0.9800
C11—H11	1.006 (16)	C20A—H20E	0.9800
C12—C13	1.391 (2)	C20A—H20F	0.9799
			
O2···C7^i^	3.22	N3···H1	2.330 (16)
O2···N1^i^	3.25	N3···H13^vii^	2.755 (17)
O2···N2	2.62	C5···C16^iii^	3.40
O2···C2^ii^	3.40	C7···C16^vi^	3.60
O2···C10^i^	3.29	C9···C14^i^	3.38
O2···N1^iii^	3.09	C10···C16^vi^	3.31
O2···C5^iii^	3.23	C11···C16^vi^	3.52
O3···C8	2.85	C11···C17^vi^	3.49
O3···C7	3.37	C1···H8B	2.547 (17)
O1···H11^iv^	2.84	C4···H20A^viii^	3.09
O2···H1^i^	2.85	C5···H20A^viii^	2.98
O2···H1^iii^	2.54	C6···H8B	2.548 (17)
O2···H2A	1.84	C8···H11	2.930 (16)
O2···H5^iii^	2.86	C11···H8A	2.688 (15)
O2···H7^i^	2.75	C13···H8B^ix^	2.895 (18)
O2···H2^ii^	2.62	C14···H8B^ix^	2.855 (17)
O3···H8A	2.25	C16···H2A	2.40
O3···H11	2.73	C17···H7^i^	2.92
O3···H12^iv^	2.69	C19···H8A	2.60
O3···H20C^v^	2.71	H1···H5	2.29 (2)
N1···O2^vi^	3.25	H1···H2A^iii^	2.50 (2)
N1···N2	2.909 (3)	H2···H2A	2.41 (3)
N1···N3	2.727 (3)	H3···H4^x^	2.57 (3)
N1···O2^iii^	3.09	H5···H20A^viii^	2.4596
N1···N2^iii^	3.078 (3)	H7···H17^vi^	2.58
N2···O2	2.62	H8A···H11	2.31 (2)
N2···C6^iii^	3.319 (3)	H8A···H20C^v^	2.50
N1···H2A^iii^	2.547 (17)	H17···H20A	2.47
			
C6—N1—C7	123.68 (11)	C14—C13—C12	118.41 (12)
C6—N1—H1	110.4 (11)	C14—C13—H13	121.5 (10)
C7—N1—H1	110.5 (11)	C12—C13—H13	120.1 (10)
C9—N2—C1	126.03 (11)	N3—C14—C13	124.02 (12)
C9—N2—H2A	114.4 (11)	N3—C14—H14	114.9 (10)
C1—N2—H2A	119.4 (11)	C13—C14—H14	121.1 (10)
C10—N3—C14	117.20 (11)	C18—O1—C19	121.73 (12)
C2—C1—C6	121.05 (12)	C9—C15—C19	119.45 (14)
C2—C1—N2	117.01 (12)	C9—C15—C16	121.02 (13)
C6—C1—N2	121.85 (11)	C19—C15—C16	119.53 (12)
C3—C2—C1	120.38 (13)	O2—C16—C17	120.18 (13)
C3—C2—H2	122.5 (10)	O2—C16—C15	123.26 (12)
C1—C2—H2	117.1 (10)	C17—C16—C15	116.50 (12)
C2—C3—C4	119.32 (13)	C18—C17—C16	121.03 (13)
C2—C3—H3	118.9 (12)	C18—C17—H17	119.5
C4—C3—H3	121.8 (11)	C16—C17—H17	119.5
C5—C4—C3	120.23 (13)	C17—C18—O1	122.28 (13)
C5—C4—H4	121.3 (10)	C17—C18—C20	126.79 (15)
C3—C4—H4	118.5 (10)	O1—C18—C20	110.93 (14)
C4—C5—C6	121.66 (13)	O3—C19—O1	114.41 (14)
C4—C5—H5	118.1 (9)	O3—C19—C15	128.34 (14)
C6—C5—H5	120.3 (9)	O1—C19—C15	117.24 (14)
N1—C6—C5	119.71 (12)	C18—C20—H20A	109.5
N1—C6—C1	122.59 (12)	C18—C20—H20B	109.5
C5—C6—C1	117.35 (11)	H20A—C20—H20B	109.5
N1—C7—C8	110.54 (10)	C18—C20—H20C	109.5
N1—C7—C10	112.50 (10)	H20A—C20—H20C	109.5
C8—C7—C10	110.48 (11)	H20B—C20—H20C	109.5
N1—C7—H7	107.7 (9)	C9—C15A—C19A	109.1 (7)
C8—C7—H7	107.9 (8)	C9—C15A—C16A	116.9 (8)
C10—C7—H7	107.5 (8)	C19A—C15A—C16A	119.9
C9—C8—C7	111.37 (11)	O2A—C16A—C17A	119.7
C9—C8—H8A	111.6 (9)	O2A—C16A—C15A	123.7
C7—C8—H8A	108.1 (8)	C17A—C16A—C15A	116.5
C9—C8—H8B	108.8 (9)	C18A—C17A—C16A	121.0
C7—C8—H8B	107.4 (9)	C18A—C17A—H17A	119.5
H8A—C8—H8B	109.4 (13)	C16A—C17A—H17A	119.5
N2—C9—C15A	117.6 (6)	C17A—C18A—O1A	122.5
N2—C9—C15	120.36 (12)	C17A—C18A—C20A	128.0
N2—C9—C8	116.04 (11)	O1A—C18A—C20A	109.5
C15A—C9—C8	125.7 (6)	C18A—O1A—C19A	121.2
C15—C9—C8	123.30 (12)	O3A—C19A—O1A	114.5
N3—C10—C11	122.76 (12)	O3A—C19A—C15A	128.0
N3—C10—C7	117.94 (11)	O1A—C19A—C15A	117.4
C11—C10—C7	119.29 (11)	C18A—C20A—H20D	109.5
C12—C11—C10	118.99 (12)	C18A—C20A—H20E	109.5
C12—C11—H11	121.2 (9)	H20D—C20A—H20E	109.5
C10—C11—H11	119.8 (9)	C18A—C20A—H20F	109.5
C11—C12—C13	118.62 (12)	H20D—C20A—H20F	109.5
C11—C12—H12	121.3 (9)	H20E—C20A—H20F	109.5
C13—C12—H12	120.0 (9)		
			
C9—N2—C1—C2	142.39 (13)	N2—C9—C15—C16	8.7 (2)
C9—N2—C1—C6	−41.15 (18)	C8—C9—C15—C16	−164.78 (12)
C6—C1—C2—C3	1.20 (19)	C9—C15—C16—O2	−8.5 (2)
N2—C1—C2—C3	177.69 (12)	C19—C15—C16—O2	171.68 (16)
C1—C2—C3—C4	−0.2 (2)	C9—C15—C16—C17	168.60 (13)
C2—C3—C4—C5	−0.7 (2)	C19—C15—C16—C17	−11.2 (2)
C3—C4—C5—C6	0.5 (2)	O2—C16—C17—C18	177.40 (14)
C7—N1—C6—C5	−130.76 (13)	C15—C16—C17—C18	0.2 (2)
C7—N1—C6—C1	56.17 (17)	C16—C17—C18—O1	7.3 (2)
C4—C5—C6—N1	−172.95 (11)	C16—C17—C18—C20	−171.50 (15)
C4—C5—C6—C1	0.47 (17)	C19—O1—C18—C17	−3.3 (2)
C2—C1—C6—N1	171.90 (11)	C19—O1—C18—C20	175.62 (14)
N2—C1—C6—N1	−4.42 (18)	C18—O1—C19—O3	171.40 (14)
C2—C1—C6—C5	−1.32 (17)	C18—O1—C19—C15	−7.8 (2)
N2—C1—C6—C5	−177.64 (11)	C9—C15—C19—O3	16.0 (3)
C6—N1—C7—C8	−16.96 (16)	C16—C15—C19—O3	−164.19 (17)
C6—N1—C7—C10	107.08 (14)	C9—C15—C19—O1	−164.88 (14)
N1—C7—C8—C9	−63.01 (14)	C16—C15—C19—O1	14.9 (3)
C10—C7—C8—C9	171.80 (10)	N2—C9—C15A—C19A	32.4 (8)
C1—N2—C9—C15A	168.4 (6)	C8—C9—C15A—C19A	−157.9 (5)
C1—N2—C9—C15	−176.24 (12)	N2—C9—C15A—C16A	172.6 (5)
C1—N2—C9—C8	−2.34 (18)	C8—C9—C15A—C16A	−17.7 (8)
C7—C8—C9—N2	76.22 (14)	C9—C15A—C16A—O2A	37.2 (8)
C7—C8—C9—C15A	−93.7 (6)	C19A—C15A—C16A—O2A	172.9
C7—C8—C9—C15	−110.09 (14)	C9—C15A—C16A—C17A	−145.4 (8)
C14—N3—C10—C11	0.16 (18)	C19A—C15A—C16A—C17A	−9.7
C14—N3—C10—C7	178.97 (11)	O2A—C16A—C17A—C18A	177.2
N1—C7—C10—N3	−5.14 (15)	C15A—C16A—C17A—C18A	−0.3
C8—C7—C10—N3	118.93 (12)	C16A—C17A—C18A—O1A	6.5
N1—C7—C10—C11	173.71 (11)	C16A—C17A—C18A—C20A	−171.8
C8—C7—C10—C11	−62.22 (15)	C17A—C18A—O1A—C19A	−2.4
N3—C10—C11—C12	−0.56 (19)	C20A—C18A—O1A—C19A	176.1
C7—C10—C11—C12	−179.35 (11)	C18A—O1A—C19A—O3A	172.4
C10—C11—C12—C13	0.40 (19)	C18A—O1A—C19A—C15A	−7.6
C11—C12—C13—C14	0.11 (19)	C9—C15A—C19A—O3A	−27.6 (8)
C10—N3—C14—C13	0.40 (18)	C16A—C15A—C19A—O3A	−166.4
C12—C13—C14—N3	−0.54 (19)	C9—C15A—C19A—O1A	152.3 (8)
N2—C9—C15—C19	−171.57 (17)	C16A—C15A—C19A—O1A	13.5
C8—C9—C15—C19	15.0 (2)		

Symmetry codes: (i) *x*, *y*+1, *z*; (ii) −*x*+1, −*y*+2, −*z*+1; (iii) −*x*+1, −*y*+1, −*z*+1; (iv) −*x*, *y*+1/2, −*z*+1/2; (v) −*x*, *y*−1/2, −*z*+1/2; (vi) *x*, *y*−1, *z*; (vii) −*x*+1, *y*+1/2, −*z*+1/2; (viii) *x*+1, *y*−1, *z*; (ix) −*x*+1, *y*−1/2, −*z*+1/2; (x) −*x*+2, −*y*+1, −*z*+1.

### Hydrogen-bond geometry (Å, º)

**Table:**

*D*—H···*A*	*D*—H	H···*A*	*D*···*A*	*D*—H···*A*
N1—H1···O2^iii^	0.889 (18)	2.536 (18)	3.089 (2)	121.0 (14)
N2—H2*A*···O2	0.928 (18)	1.836 (18)	2.616 (2)	140.1 (15)
N2—H2*A*···O3*A*	0.928 (18)	1.58 (2)	2.382 (15)	142.0 (17)

Symmetry code: (iii) −*x*+1, −*y*+1, −*z*+1.
